# Glycation modulates alpha-synuclein fibrillization kinetics: A sweet spot for inhibition

**DOI:** 10.1016/j.jbc.2022.101848

**Published:** 2022-03-18

**Authors:** Azad Farzadfard, Annekatrin König, Steen Vang Petersen, Janni Nielsen, Eftychia Vasili, Antonio Dominguez-Meijide, Alexander K. Buell, Tiago Fleming Outeiro, Daniel E. Otzen

**Affiliations:** 1 Interdisciplinary Nanoscience Center (iNANO), Aarhus University, Aarhus C, Denmark; 2 School of Biology, College of Science, University of Tehran, Tehran, Iran; 3 Experimental Neurodegeneration, University Medical Center Göttingen, Göttingen, Germany; 4 Department of Biomedicine, Aarhus University, Aarhus C, Denmark; 5 Laboratory of Neuroanatomy and Experimental Neurology, Department of Morphological Sciences, Center for Research in Molecular Medicine and Chronic Diseases (CIMUS), Instituto de Investigación Sanitaria de Santiago de Compostela (IDIS), University of Santiago de Compostela, Santiago de Compostela, Spain; 6 Department of Biotechnology and Biomedicine, Technical University of Denmark, Lyngby, Denmark; 7 Max Planck Institute for Natural Sciences, Göttingen, Germany; 8 Faculty of Medical Sciences, Translational and Clinical Research Institute, Newcastle University, Newcastle Upon Tyne, United Kingdom; 9 Scientific Employee with an Honorary Contract at German Center for Neurodegenerative Diseases (DZNE), Göttingen, Germany; 10 Department of Molecular Biology and Genetics, Aarhus University, Aarhus C, Denmark

**Keywords:** Parkinson's disease, glycation, neurodegenerative diseases, alpha-Synuclein, αSN, alpha-synuclein, AGEs, advanced glycation end products, CD, circular dichroism, MGO, methylglyoxal, PD, Parkinson’s disease, PTMs, posttranslational modifications, ThT, Thioflavin T

## Abstract

Glycation is a nonenzymatic posttranslational modification (PTM) known to be increased in the brains of hyperglycemic patients. Alpha-synuclein (αSN), a central player in the etiology of Parkinson’s disease, can be glycated at lysine residues, thereby reducing αSN fibril formation *in vitro* and modulating αSN aggregation in cells. However, the molecular basis for these effects is unclear. To elucidate this, we investigated the aggregation of αSN modified by eight glycating agents, namely the dicarbonyl compound methylglyoxal (MGO) and the sugars ribose, fructose, mannose, glucose, galactose, sucrose, and lactose. We found that MGO and ribose modify αSN to the greatest extent, and these glycation products are the most efficient inhibitors of fibril formation. We show glycation primarily inhibits elongation rather than nucleation of αSN and has only a modest effect on the level of oligomerization. Furthermore, glycated αSN is not significantly incorporated into fibrils. For both MGO and ribose, we discovered that a level of ∼5 modifications per αSN is optimal for inhibition of elongation. The remaining sugars showed a weak but optimal inhibition at ∼2 modifications per αSN. We propose that this optimal level balances the affinity for the growing ends of the fibril (which decreases with the extent of modification) with the ability to block incorporation of subsequent αSN subunits (which increases with modification). Our results are not only relevant for other αSN PTMs but also for understanding PTMs affecting other fibrillogenic proteins and may thus open novel avenues for therapeutic intervention in protein aggregation disorders.

Glycation (also known as the Maillard reaction or nonenzymatic glycosylation) refers to a cascade of reactions between ketones or aldehydes (found in reducing sugars and other metabolites) and amino groups (1). This starts with the formation of Amadori compounds and leads, *via* the formation of various intermediates, to the accumulation of a collection of advanced glycation end products (AGEs) (2, 3, 4). Due to its nonenzymatic origin (which contrasts with other posttranslational modifications (PTMs) such as phosphorylation, acetylation, or methylation), glycation does not occur at defined loci or time points. Proteins can be modified at the N terminus, Arg guanidinium groups, or Lys amino groups (5, 6), while nucleic acids and lipids are glycated *via*, *e.g.*, the free amino group of guanine (7) or phosphoethanolamines (8), respectively. While reducing sugars such as ribose and glucose can play a role in these processes, the most important glycating agents found *in vivo* are low-molecular-weight carbonyl compounds (9), in particular the dicarbonyl metabolite methylglyoxal (MGO, Fig. 1*A* insert) (10, 11) that is generated as a byproduct of glycolysis.

**Figure 1:**
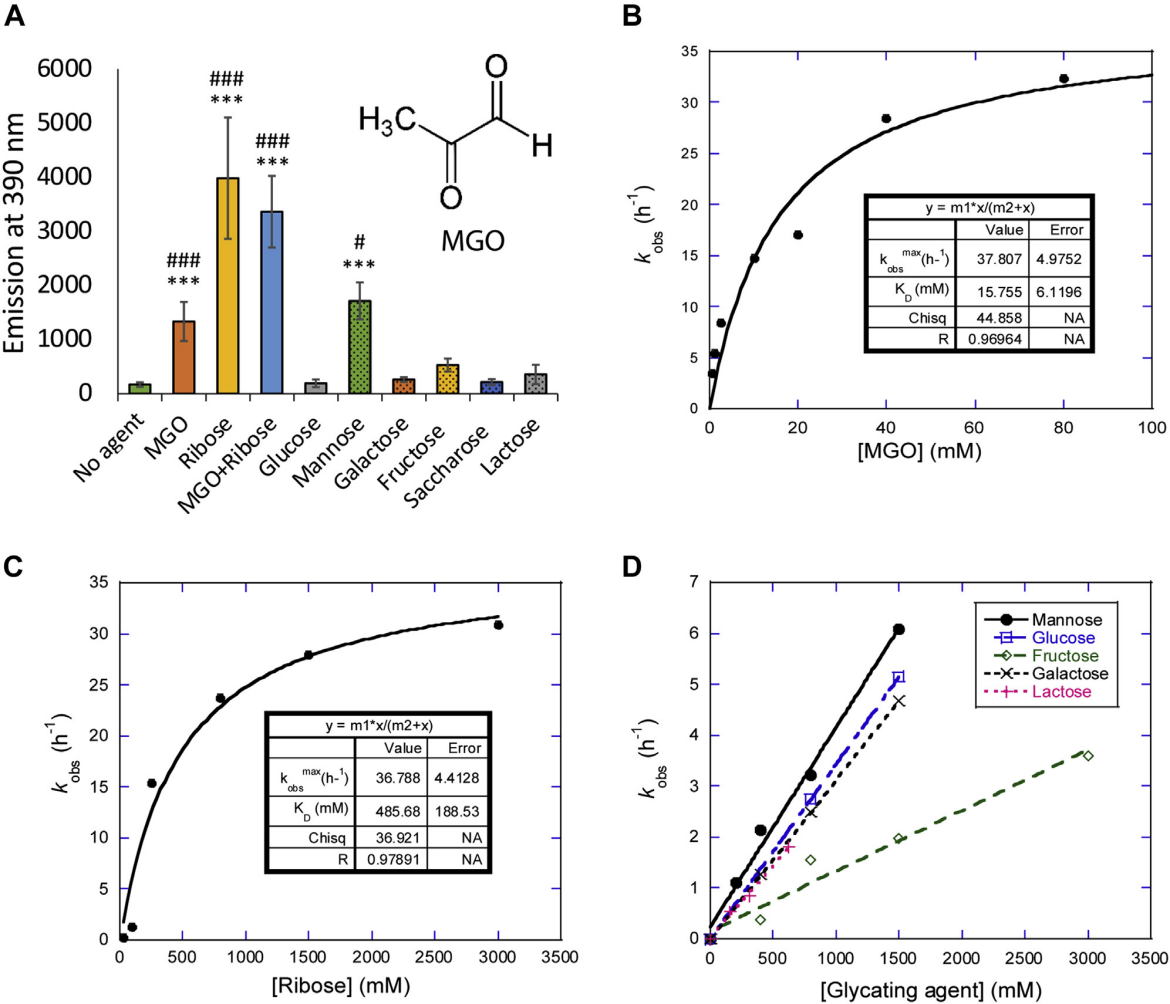
**αSN is glycated to different extents by different compounds *in vitro*.***A*, 100 μM αSN was incubated alone or with different glycating agents (5 mM MGO, 0.8 M ribose, 1 M glucose, 1 M mannose, 0.25 M galactose, 2 M fructose, 1.25 saccharose, 0.5 M lactose) for 5 days at 37 °C under constant agitation (300 rpm). Fluorescence of glycation products was measured by excitation at 240 nm and emission at 390 nm. Error bars display standard deviation. Statistical significance was assessed *via* ANOVA with a post hoc Tukey. #: compared to respective reagent w/o αSN (see Fig. S1 for graph), ∗: compared to αSN incubated without glycating agent. ^#^*p* < 0.05, ^###^*p* < 0.0001, ∗∗∗*p* < 0.0001. *B*–*D*, kinetic rate constants of glycation obtained for MGO (*B*) and ribose (*C*), and initial rates of glycation measured for other modifiers (*D*) based on data shown in Fig. S2. Data in panels *B* and *C* fitted to a binding isotherm. Note that initial rates were normalized according to the extent of covalent modification (see main text). αSN, α-synuclein; MGO, methylglyoxal.

Hyperglycemia, as frequently observed in diabetes, causes complications associated with protein glycation but is also associated with various neurodegenerative diseases (see below). Glycated proteins not only induce inflammation through the AGE receptors on cell surfaces but also affect protein structure and function by altering the chemical composition of the modified residues (12, 13, 14). For instance, albumin glycation (an inevitable consequence of high blood sugar levels) induces molten globule states (15), and these destabilized states can more easily form amyloid-like fibrils, which in turn can act as seeds for aggregation-prone proteins like amyloid-beta (16).

The intrinsically unfolded protein Alpha-synuclein (αSN) has a central role in the etiology of Parkinson’s disease (PD) and other diseases, collectively termed synucleinopathies. Some of these diseases are known for the accumulation of intracellular protein inclusions known as Lewy bodies and Lewy neurites, composed, among other proteins and molecules, of aggregated αSN (17). αSN overexpression or mutations cause rare genetic forms of PD. Importantly, the most common form of PD is sporadic, without apparent changes in the levels of expression of WT αSN (18). Nevertheless, PD is thought to be associated with the tendency of αSN to form oligomeric and amyloid-like aggregates (19). While a definite causal relationship between the type of αSN aggregation and neurodegeneration remains controversial (20), it is thought that certain types of aggregated αSN species are toxic to neurons. In particular, soluble intermediate species (oligomers) are more toxic on a per-mass level than mature fibrils (21, 22, 23). Glycation of αSN and AGE adducts has been described in the brains of PD patients, particularly the frontal cortex (24, 25). AGEs have also been detected in Lewy bodies in pre-PD stages and are increased in αSN, purified from PD patients’ erythrocytes (26, 27).

αSN is glycated by MGO as well as by reducing sugars such as ribose and glucose. While the glyoxalate systems with the glyoxalases Glo1 and Glo2 catabolize reactive dicarbonyls in cells, it is thought that once early and advanced glycation products are formed, they become rather stable (2, 28). Since αSN is a long-lived protein with 15 lysine residues in its primary amino acid sequence, it can potentially be glycated at multiple sites over time, leading to the accumulation of a variety of early glycation products and AGEs (12, 29). These modifications compromise αSN homeostasis (30, 31) through impaired membrane binding, altered aggregation behavior, localization, clearance *via* a reduction in αSN ubiquitination and SUMOylation, and increased intercellular propagation because of reduced extracellular protease cleavage (30, 31). Moreover, glycation of aSN can result in inactivation of glyceraldehyde 3-phosphate dehydrogenase, a glycolytic enzyme associated with neurodegenerative diseases (32), further emphasizing the harmful effects of αSN glycation (33). Several antidiabetic drugs have beneficial effects in PD patients, but the precise molecular mechanisms underlying these effects are unknown. Recently, it was found that DJ-1 (linked to familial PD) has deglycase activity, removing MGO and glyoxal from αSN and alleviating the effects of MGO-induced aggregates of αSN in cells (34, 35, 36).

Glycation directly affects αSN aggregation behavior, promoting the accumulation of intermediate species at the expense of fibril formation (31, 37, 38). Thus, it is tempting to hypothesize that glycation enhances PD-associated αSN pathology. Glycation-induced changes in aggregation is not unique for αSN (39) and has been shown to promote (40, 41, 42) and slow down the fibrillation of amyloid-beta (43, 44). Importantly, glycated monomeric or oligomeric αSN not only does not form fibrils on its own but also prevents fibril formation of unmodified αSN. However, the molecular mechanism involved in these effects on aggregation remains unclear. Here, we examine the effects of eight different naturally occurring glycation agents on modification and fibril formation of αSN, namely MGO, the pentose sugar ribose, and the hexose sugars fructose, mannose, glucose, galactose, sucrose, and lactose.

Glucose is the key energy source in living organisms and present in the millimolar range in human plasma. Ribose is a highly abundant naturally occurring sugar (100 μM in fasting serum) that is essential for every living cell, since it is a component of several biomolecules including RNA, DNA, and ATP (45). Fructose, a common supplement in the typical Western diet, has received much attention in the last years. Human fasted serum fructose levels are in the micromolar range and are elevated in diabetic patients (46). Lactose is the primary energy source in the first months of human life. In addition, we completed our systematic approach by including mannose, galactose, and the disaccharide sucrose. Mannose is a monosaccharide required for glycoprotein synthesis. The serum concentration of mannose is approximately 40 μM (47). Galactose, one of the most abundant sugars in human adult diet, is important in many metabolic processes, including glycosylation (48).

MGO is clearly the most reactive species, followed by ribose, while the six other sugars show much lower levels of reactivity. This reactivity ranking is maintained when we examine the impact on aggregation of αSN. We find that glycated αSN strongly inhibits the fibril elongation step but not the initial nucleation step. The highest level of inhibition of unmodified αSN is achieved using αSN modified with around 4 to 5 MGO or ribose groups per αSN, while the remaining sugars inhibit more weakly, with their effects peaking at 2 to 3 sugars per αSN. We propose that this optimal level is caused by a trade-off between reduced binding affinity for the growing fibril ends with glycation and the increased ability to block elongation. We also suggest that this will have relevance for other types of PTMs, opening novel concepts for intervention in protein aggregation disorders.

## Results

### MGO and to a smaller extent ribose efficiently glycate αSN

We started out by measuring the extent to which different agents glycate αSN. Recombinant human αSN was incubated with each glycating agent for 5 days at 37 °C, with constant agitation. Since several glycation products fluoresce, we monitored glycation through the increase in fluorescence at their maximum intensity at 390 nm when excited at 240 nm (Fig. S1*A*) (15, 49). There was a large difference between different agents. MGO, ribose, and mannose induced a significant increase in fluorescence intensity, while the remaining five hexose sugars did not (Fig. 1*A*). The glycating agents alone were not fluorescent (Fig. S1*B*). We note that neither glycating agents nor glycated αSN are expected to interfere with the fluorescence of Thioflavin T (ThT) (excitation at 450 nm and emission at 485 nm) even after excitation at 438 nm (Fig. S1*C*).

To investigate the kinetics of αSN glycation, we monitored the fluorescence signal over time in a plate reader over a week at 37 °C, using different concentrations of glycating agent. To minimize aggregation, samples were incubated without shaking. We subsequently confirmed, by centrifugation and A_280_ analysis of the supernatant of unmodified αSN, that the amount of soluble αSN was unchanged at the end of the incubation period. Furthermore, analysis by ThT fluorescence showed no fibril formation (data not shown). The fluorescence signal associated with the glycation of αSN by MGO and ribose followed an inverted exponential decay over time, while the fluorescence induced by other glycating agents showed a linear increase, indicating that the kinetics for these agents was too slow to reach a plateau within the 6 to 8 days of the experiment (Fig. S2). Rate constants obtained from the exponential decays with MGO and ribose showed a hyperbolic dependence on the concentration of glycating agent, indicating a saturation phenomenon analogous to that of the Michaelis–Menten curve in enzyme kinetics (Fig. 1, *B* and *C*). From these curves, we estimated apparent binding constants of 15 mM and 486 mM for MGO and ribose, respectively (Table 1). For the less efficient glycating agents, we only observed a linear relationship between the slopes of the plots in Fig. S2 and the concentration of the agent (Fig. 1*D*). Importantly, different glycating agents do not produce exactly the same fluorescent AGE products when reacting with Lys or His residues on αSN, precluding direct comparison of the raw fluorescence data (49, 50, 51). This was also apparent in our data. When data from all agents were compared, changes in the fluorescence signal obtained with different glycating agents did not correlate directly with the changes in the extent of αSN modifications according to MALDI-TOF MS (Fig. S3). Fortunately, for each individual agent, the fluorescence signal correlated with the extent of modification. Therefore, we normalized the fluorescent data according to the extent of glycation at the end-stage of incubation for each glycating agent to be able to compare the glycation rates in different agents. To allow direct comparison between the two most efficient glycating agents, we analyzed the raw data from these modifications in the same manner as that for the less reactive agents, *i.e.*, we determined the initial slopes of the time course of glycation by MGO and ribose from Fig. S2 and plotted them *versus* [MGO] and [ribose]. The slope of the initial linear part of that plot can be compared directly with the values for the other glycation agents (Table 1). Clearly, MGO was the most efficient agent, followed by ribose which shows a 30-fold lower affinity and a 32-fold lower initial rate constant. Then, there was a large gap down to the less reactive group, ranked in decreasing reactivity: mannose> glucose > galactose > lactose > fructose. Finally, sucrose had undetectable glycating activity.

**Table 1:** Kinetic parameters for αSN glycation^a^

Binding mode	Glycating agent	*K*_D_ (mM)^b^	*k*_max_ (h^−1^)^b^	Initial slope (h^−1^M^−1^)^c^
Saturation binding	MGO	15.8 ± 6.12	37.8 ± 4.98	2392
	Ribose	486 ± 188	36.8 ± 4.41	75
Linear^c^	Mannose	-	-	3.91 ± 0.26
	Glucose	-	-	3.43 ± 0.00
	Galactose	-	-	3.10 ± 0.01
	Lactose	-	-	2.81 ± 0.16
	Fructose	-	-	1.19 ± 0.13
	Sucrose	-	-	No detectable signal

a All data based on fluorescence time profiles normalized by the extent of modification (see main text).

b The rate constant for glycation *k*_obs_ was obtained from exponential decays in Figure 1, *B* and *C*. Plots of *k*_obs_*versus* glycating agent concentration were then fitted to a Michaelis–Menten type model to obtain the apparent affinity of the agent for αSN (*K*_D_) and the maximal velocity at high agent concentrations (*k*_max_).

c Initial velocities at every glycating agent concentration was obtained from normalized slopes *versus* time at time *t* = 0. The table provides the slope obtained from plotting initial velocities *versus* glycating agent concentration (in the case of MGO and ribose, whose glycation profile followed an exponential decay, the slope was obtained as the tangent at zero concentration).

### Glycation does not change αSN structure or induce αSN oligomerization

**Figure 2:**
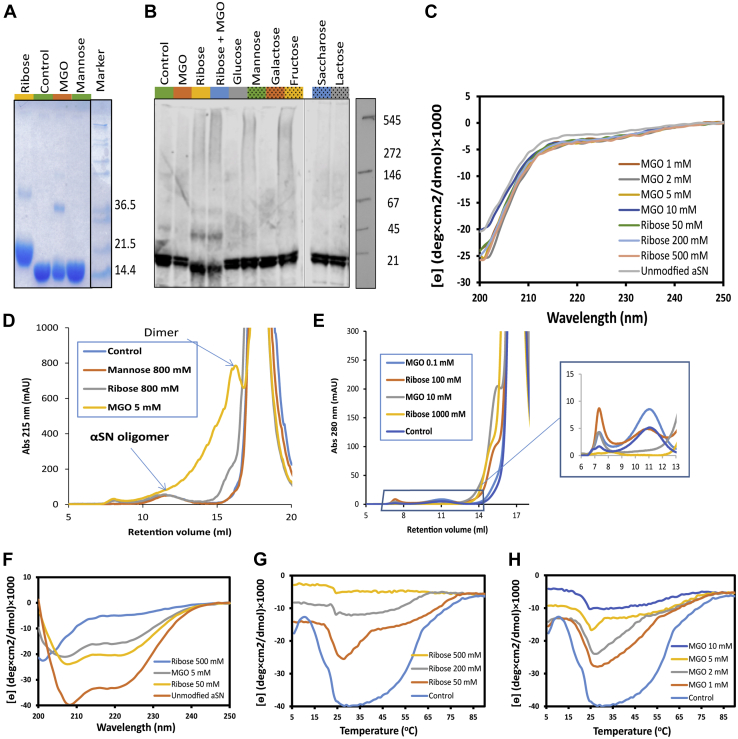
**Size and structure of different species of glycated αSN.***A*, SDS-PAGE analysis of αSN glycated by incubation with the indicated agents. Molecular weights of different bands are indicated. *B*, αSN glycated by incubation with the indicated agents as indicated in Figure 1*A* was analyzed *via* immunoblot under nondenaturing conditions. Primary antibody used is anti-αSN1. *C*, far-CD spectra show a random coil structure for all the glycated aSN similar to control. *D*, SEC shows same oligomer peak (eluting around 12 ml) for all glycated modified αSN. In addition, a substantial dimer peak and other paucimeric species are observed for MGO-modified αSN, as well as a small amount of dimer of ribose-modified αSN. *E*, oligomer formation of glycated αSN is suppressed after glycation with 1000 mM ribose or 10 mM MGO for 9 days incubation in 37 °C followed by purification of monomeric αSN. Glycation was carried out using monomeric αSN purified by size-exclusion chromatography, and oligomer formation was then induced by three cycles of freeze-drying of the sample in MQ water. *F*, far UV CD of unmodified and glycated aSN in the presence of 1 mg/ml DMPG at 25 °C. Highly ribosylated αSN (500 mM ribose) shows no change in its secondary structure, indicating no interaction with vesicles. MGOylated and less ribosylated αSN (50–200 mM ribose) show more modest changes in secondary structure however compared to unmodified αSN. *G* and *H*, thermal transition between random coil and α-helix by glycated αSN measured by ellipticity at 222 nm during a temperature ramp from 6 to 90 °C in the presence of 1 mg/ml DMPG. The greater the extent of glycation of αSN, the smaller the transition signal. αSN, α-synuclein; CD, circular dichroism; MGO, methylglyoxal; SEC, size-exclusion chromatography.

At the end of the glycation reaction, excess glycating agent was removed by desalting to enable subsequent analyses of the modified αSN. Using SDS-PAGE, we found a dominant monomeric band. In addition, we found that ribose and MGO gave rise to a weaker band corresponding to an αSN dimer. The band corresponding to monomeric ribose–αSN was slightly shifted upward, indicating extensive modification (Fig. 2*A*). Interestingly, in native gel electrophoresis, ribose-treated αSN migrated slightly further than the other αSN samples, suggesting a slight increase in protein compaction (Fig. 2*B*). However, circular dichroism (CD) spectroscopy did not show any substantial differences in secondary structure, indicating that αSN remained disordered (Fig. 2*C*), and small angle X-ray scattering studies did not show any differences in overall conformation between differently modified αSN preparations (data not shown).

Using a filter trap assay, we found that MGO-treated αSN was not retained on a 0.2 μm acetate membrane, in contrast to untreated αSN and ribose-treated αSN (Fig. S4*A*), indicating that MGO species remained largely soluble, in contrast to those present in the other two samples. Using size exclusion chromatography to assess the size of the soluble species formed as a result of glycation, we observed a peak (MGO) or shoulder (ribose) preceding the monomer peak (Fig. 2*D*). No such bands or peaks were seen for other glycating agents. The MGO dimer peak was preceded by a broad shoulder eluting between ca. 11 and 15 ml (Fig. 2*D*), suggesting the formation of a range of species between dimer and oligomer (the oligomer elutes at 10–13 ml). There were also faint hints of trimers and higher-order MGO-αSN species, as determined by SDS-PAGE (Fig. 2*A*). Collectively, we name these smaller species *paucimers* (Latin paucus: few) to differentiate them from the larger (∼30-mer) αSN *oligomers*.

The oligomers observed were formed in a one-pot reaction where glycation and oligomerization of unmodified αSN could occur in parallel (Fig. 2*D*). To separate the two processes, we first purified highly glycated αSN by size exclusion chromatography and then subjected these monomeric preparations to oligomerization. Normally only 1 to 5% of αSN is converted into oligomers after our incubation protocol, but we have recently discovered that multiple rounds of freeze-drying increase the yield of oligomeric αSN (A.F. and D.E.O., unpublished observations). However, glycated monomeric αSN failed to form significant amounts of oligomers, even after three rounds of freeze-drying (Fig. 2*E*).

### Glycation strongly reduces αSN interactions with anionic membranes

We next investigated how glycation affected the interaction of αSN with membranes. Monomeric αSN is known to bind phospholipid membranes, a property which is likely closely linked to its biological function (52). The protein has particularly high affinity for anionic lipids, and the contact is largely mediated by the N-terminal domain, which is rich in lysine (Lys) residues (53). This process can be monitored *in vitro* using synthetic liposomes made of anionic lipids such as DMPG (54), since binding induces α-helical structure which can be detected by far-UV CD. A thermal scan of the αSN–DMPG mixture leads to a characteristic biphasic transition. At temperatures below 20 °C, αSN shows very little helicity, but subsequent heating gives rise to a large signal increase which stabilizes around 25 °C as DMPG transitions to the liquid disordered phase and αSN undergoes “warm refolding” (the reverse of cold denaturation). At higher temperatures, the CD signal melts out as interactions with the membrane weaken (54). Glycation has a striking impact on this. Heavily ribose-modified αSN did not show any helical structure in the presence of DMPG unlike unmodified αSN (Fig. 2*F*) and hardly underwent any thermal transition (Fig. 2, *G* and *H*). The greater the modification, the smaller the thermal response observed. This was the case both for ribose- and MGO-modified αSN. Thus, modification of Lys residues had strong inhibitory effects on the interactions of αSN with anionic membranes.

### Glycated αSN reduces the elongation rate during fibril formation of unmodified αSN

**Figure 3:**
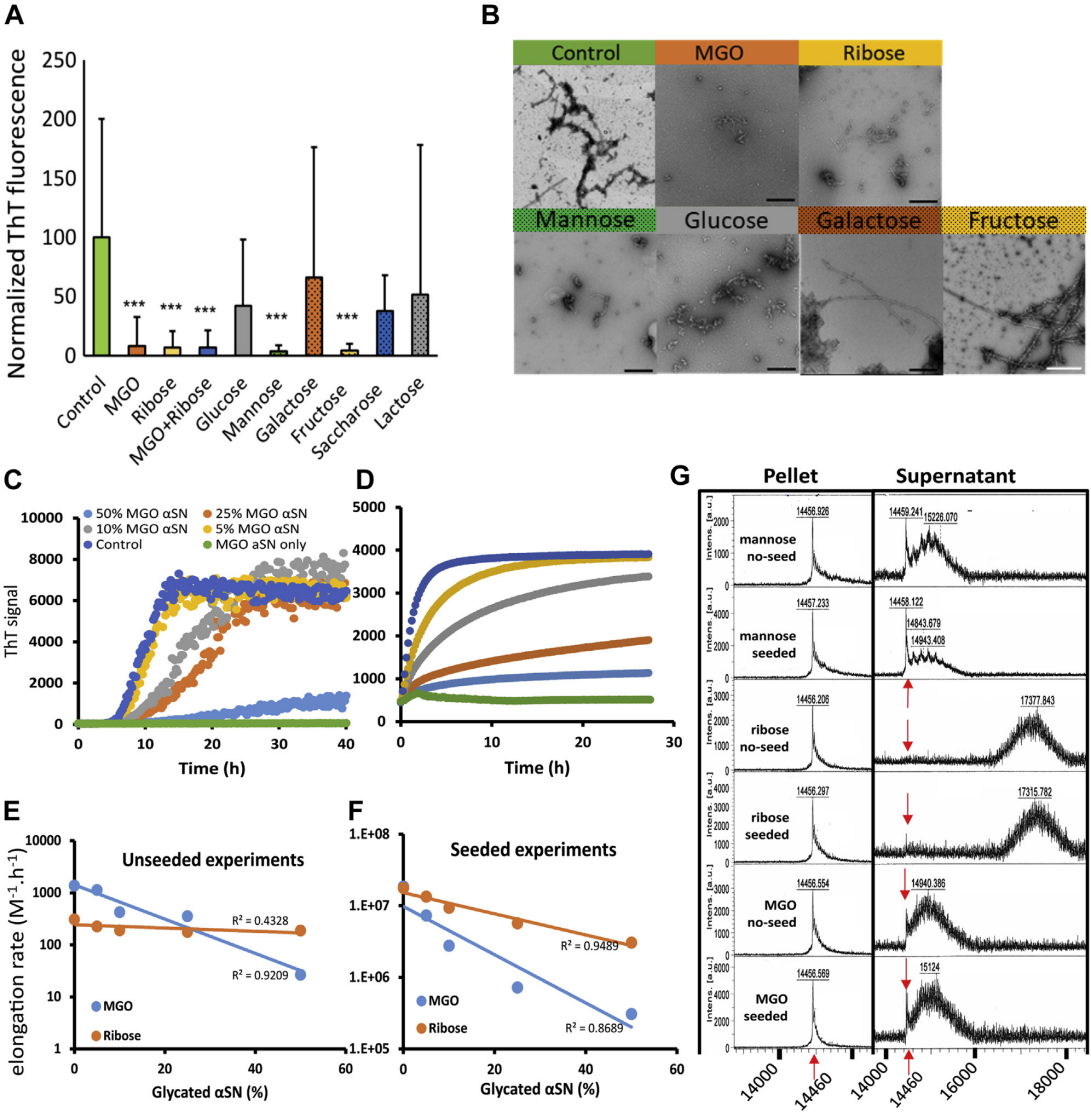
**Glycated αSN reduces the fibrillation rate of unmodified αSN.***A*, 10 μg/ml of monomeric unmodified αSN was incubated with 1 μg/ml of glycated αSN (the latter prepared as in Fig. 1*A*) in cycles of repeated shaking and incubation for 48 h, after which ThT fluorescence was measured. Statistical significance was assessed *via* ANOVA with a post hoc Tukey. Data were normalized to control (100%, αSN incubated without glycating agent). ∗∗∗*p* < 0.0001. *B*, TEM images of fibrils obtained in part A. Scale bar: 200 nm. *C* and *D*, fibrillation kinetics of unmodified αSN in the presence of 0 to 0.5 mg/ml MGO-αSN (*i.e.*, 0–50% of monomer) without seed (*C*) and with 5% seed (*D*). *E* and *F*, elongation rates obtained from Amylofit analysis decay approximately exponentially with the amount of added glycated αSN. *G*, MALDI-TOF MS analysis of αSN found in the precipitant (*left panel*) and supernatant (*right*) fraction of the fibrillation experiments in *C* and *D*. All glycated αSN is found in the supernatant (nonfibrillated) fraction. Fibrils in the precipitant fraction were dissociated in 10% formic acid, and the formic acid was removed by freeze-drying before MS. αSN, α-synuclein; MGO, methylglyoxal; ThT, Thioflavin T.

We then assessed the effect of glycation on the fibrillization of αSN under constant agitation where unmodified αSN monomers are known to readily form amyloid fibrils. The samples were spiked with 10% of the αSN preparations characterized in Figure 1*A*. In the sample that had been spiked with the unmodified αSN preparation, ThT-positive species were formed. In contrast, we observed that spiking with αSN modified by ribose, MGO, mannose, or fructose strongly inhibited fibrillization in the samples over 48 h incubation at 37 °C (Fig. 3*A*). A similar effect was observed in a previous study of the aggregation kinetics of αSN monomers treated with MGO (31). Transmission electron microscopy revealed that especially the samples spiked with MGO- and ribose-modified αSN do not show the typical fibrillar structures (Figs. 3*B* and S4*B*). Furthermore, we found that αSN species of intermediate size formed after 24 h upon treatment with ribose or MGO-modified αSN. The characteristic fibrils that formed in control preparations (*i.e.*, without addition of pre-incubated αSN) only emerged after several days of constant agitation (Fig. S4*B*).

To investigate the basis for the observed inhibitory effect, we prepared MGO-modified αSN by incubating αSN with 5 mM MGO for 5 days under quiescent conditions, in order to favor the glycation reaction with respect to aggregation. This treatment led to ca. seven MGO groups per αSN, according to MALDI-TOF MS. We then added 0.05 to 0.5 mg/ml of this MGO-αSN to 1 mg/ml of unmodified αSN, both in the absence or presence of 50 μg/ml (5%) seeds of preformed αSN fibrils (from unmodified αSN). In the absence of seeds, MGO-αSN strongly impeded the growth phase (the slope was reduced 20-fold by 50% MGO-αSN), while the lag phase was only ca. 2-fold increased (Fig. 3*C*). On its own, MGO-αSN did not form fibrils. Seeding bypasses the need for nucleation, making elongation of the seed fibrils the rate limiting step in the overall conversion into fibrils. In this case, MGO-αSN also strongly reduced elongation (Fig. 3*D*). To gain further mechanistic insight, the data were analyzed with different aggregation models using the webserver AmyloFit (55). The best fit was obtained using a model with a secondary process and elongation. We know from a series of prior studies that under the conditions of these experiments (neutral pH, mechanical agitation), the dominant secondary process is fibril fragmentation (56, 57). Amylofit provides a better fit for unseeded kinetics curves when the rate of elongation (*k*_*+*_) or the rate of fragmentation (*k*_−_), rather than the rate of primary nucleation (*k*_n_), is varied (Fig. S5, *A*–*C*). In strongly seeded experiments, we largely bypass nucleation, and even fragmentation cannot manifest itself significantly, leaving elongation as the dominant mechanistic step. For the seeded curves, varying only *k*_+_ provided the best fits (Fig. S5, *D*–*F*), while varying both *k*_+_ and *k*_−_ at the same time did not improve fits further (data not shown). The elongation rates obtained in Amylofit analysis decrease in an approximately exponential fashion with the percentage of modified αSN, leading to a ∼50-fold reduction in *k*_+_ between 0 and 50% modified αSN for both unseeded and seeded experiments (Fig. 3, *E* and *F*). This finding strongly suggests that MGO-αSN mainly inhibits the elongation step of unmodified αSN fibril formation. In contrast, neither ribose-modified αSN (containing 15 ribose units per αSN) nor mannose-modified αSN (with two mannose per αSN) were able to suppress the fibrillization with or without seeds as efficiently as MGO-modified αSN (Fig. S6, *A*–*D*). Thus, ribose only leads to a ∼5-fold decrease in *k*_+_ between 0 and 50% modified αSN. See below for further analysis of the impact of the level of glycation on the inhibition of fibril formation. Neither MGO- nor ribose-modified αSN were able to form fibrils on their own or in seeded reactions (Fig. S6, *E* and *F*), whereas mannose-modified αSN fibrillated almost as well as unmodified αSN, due to its low level of glycation. As an additional control, we confirmed that MGO-modified albumin did not affect the fibril formation of unmodified αSN (Fig. S6*G*). Thus, AGE products do not interfere with fibrillization by themselves, but need to be located on αSN.

We next investigated the extent to which different types of modified αSN were incorporated into fibrils. After fibrillization (both seeded and unseeded), αSN was separated into fibrils (pellets) and soluble species (the supernatant) by centrifugation. Remarkably, modified αSN was not detected in the fibril fraction but was entirely confined to the supernatant (Fig. 3*G*). This was uniformly seen for modification by either MGO, ribose, or mannose. Thus, glycated αSN actively interferes with the fibrillization process without being stably incorporated into fibrils.

### Both type and level of glycation affect the elongation rate of unmodified αSN fibrils

**Figure 4:**
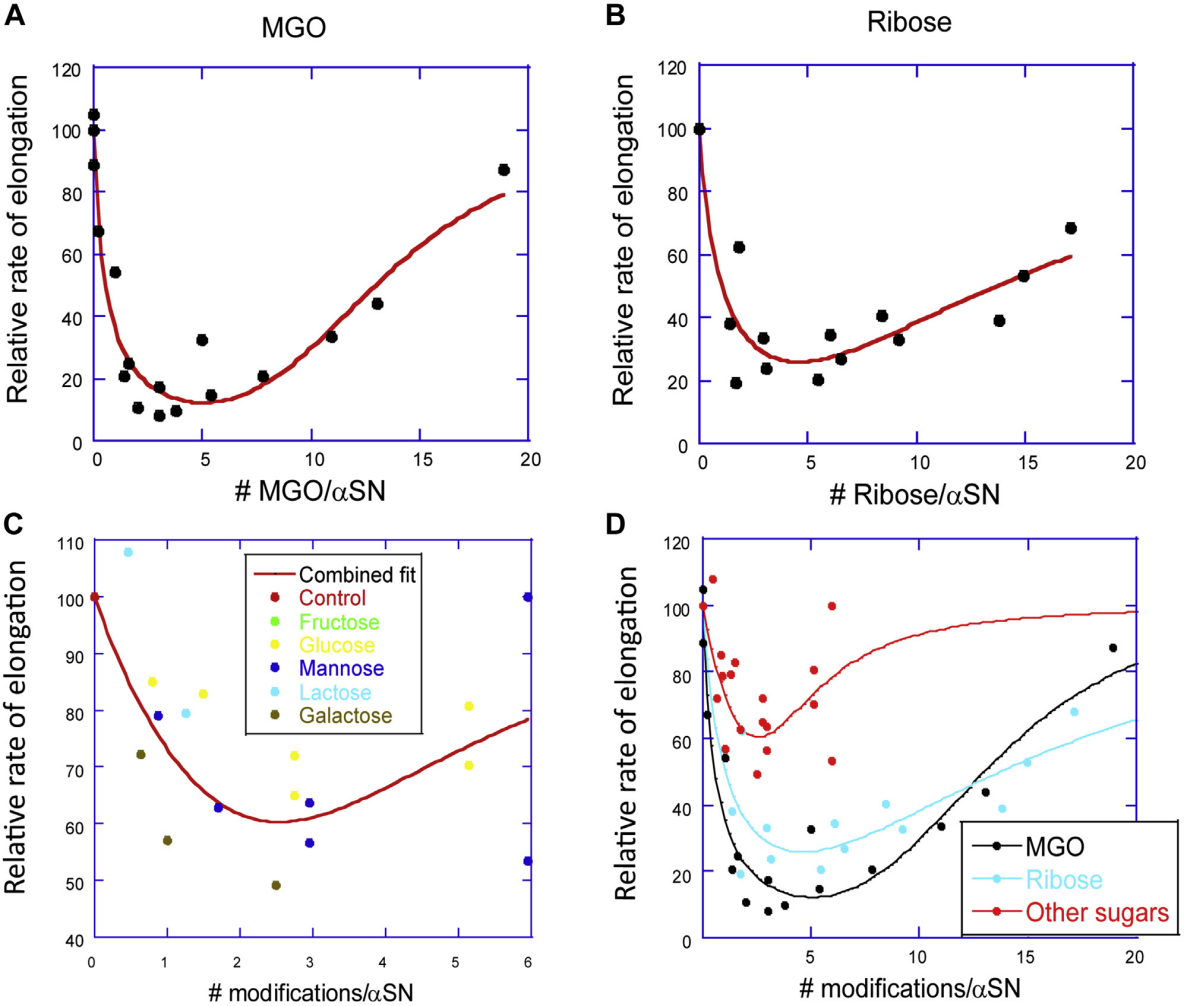
**Effect of type and extent of glycation on elongation rate.** All types of glycated αSN show maximum efficiency in decreasing the elongation rate when ∼5 residues per αSN molecule are modified. MGO (*A*) and ribose (*B*) reduce the elongation rate most effectively, whereas the other modifiers (*C* and *D*) are less effective. In *D*, all data in panel *C* are combined into the “Other CHO” fraction. The initial rate of elongation was obtained from a seeded ThT fibrillation assay relative to control. The extent of glycation for each sample was determined by MALDI-TOF MS. Joined lines are best fits of Equation 2 to the data. αSN, α-synuclein; MGO, methylglyoxal; ThT, Thioflavin T.

We hypothesized that the ability of modified αSN to inhibit αSN fibrillization was a balance between two opposing effects: modified αSN has to be able to bind to the fibrils’ growing ends to a reasonable extent to compete with unmodified αSN monomers (step 1), while blocking the addition of unmodified αSN to the terminally bound modified αSN (step 2). Increasing levels of modification would disfavor step 1 but favor step 2. This competition of effects predicts an optimal level of glycation which may vary from one glycation type to another. This prompted us to investigate how the average level of glycation affected the fibrillization of unmodified αSN. For this, αSN was first glycated in the presence of different concentrations of different glycation agents. We then used MALDI-TOF MS to measure the average mass of glycated αSN and obtained the average degree of glycation by dividing the increase in mass by the mass of the individual sugars. We subsequently carried out seeding assays using 0.5 mg/ml unmodified αSN and 0.1 mg/ml of αSN glycated to different extents. Each fibrillization curve was fitted according to equation y(t) = Amp[1 − exp(−kt)], where y(t) is ThT signal at the time t, Amp is the amplitude of the reaction, and *k* the rate constant. The initial velocity (*i.e.*, elongation at time zero) was obtained as the value of the first derivative at time zero (=Amp∗*k*). In agreement with our hypothesis, we identified an optimal level of glycation which led to the greatest reduction in fibrillization (Fig. 4). For both ribose and MGO, this occurred when αSN was modified with around five glycation molecules per αSN. For visualization purposes, the impact of these two counterbalancing effects was illustrated by fitting the data to a simple model for inhibition and cancellation of inhibition, inspired by a model for the activation and deactivation of lipase activity by multiple surfactant binding steps (58):

(1) $$\text{αSN}+\text{CHO}⬄\text{αSN:CHO}⬄\text{α}{\text{SN:CHO}}_{n+1}$$

where CHO represents a glycation group and *n* is a number. The first binding equilibrium (with a formal dissociation constant *K*_1_ = [αSN][CHO]/[αSN:CHO]) corresponds to the initial modification of αSN which leads to a reduction in fibril formation since it blocks step 1 (binding of modified αSN) less than step 2 (binding of unmodified αSN). The second binding equilibrium (with *K*_2_ = [αSN:CHO][CHO]^*n*^/[αSN:CHO]^*n+1*^) corresponds to additional modification steps (*n* > 1 to indicate that more modifications occur at this stage than in the first stage) where step 1 is blocked more than step 2. Obviously, covalent modifications such as glycation do not correspond directly to binding equilibria but translate into changes in binding affinities of αSN to the growing ends of the fibrils. By formally treating this as a transition between three states αSN, αSN:CHO, and αSN:CHO_*n+1*_ with three different levels of elongation capacities and using the average level of glycation as the x-variable denoting the formal concentration of the variable CHO, we obtain an equation to describe how the observed relative elongation rate *v*_elongation_ varies with the level of glycation:

(2) $$v=\frac{v_{aSN}*\frac{K_{1}}{\left[CHO\right]}+v_{aSN:CHO}+v_{aSN:CHO_{n+1}}*\frac{{\left[CHO\right]}^{n}}{K_{2}}}{\frac{K_{1}}{\left[CHO\right]}+1+\frac{{\left[CHO\right]}^{n}}{K_{2}}}$$

K_1_ and K_2_ should then be regarded as probabilities of modification. In practice, it is simplest to set *v*_αSN_ = *v*_αSN:(CHO)(n+1)_ = 100 and *v*_αSN:CHO_ = 0 (the counterbalancing effects of the two formal binding equilibria mean that it is difficult to obtain a meaningful value for *v*_αSN:CHO_ if it is allowed to vary freely). As shown in Figure 4, *A* and *B*, the model described the data satisfactorily and captures the existence of a broad minimum centered around five glycation units per αSN molecule. Despite the obvious limitations and simplifications in the model, the data allowed us to provide a semiquantitative estimate of the impact of the two modifications on elongation *via* the associated *K*_1_ and *K*_2_ values (Table 2) as well as estimate the lowest level of elongation.

**Table 2:** Parameters describing the impact of glycation on the elongation of αSN

Glycation agent	*K*_1_^a^	*n*^b^	*K*_2_^1/n^(# modifications)^c^
MGO	0.56 ± 0.11	3.67 ± 1.07	13.1
Ribose	1.03 ± 0.28	1.84 ± 0.68	14.3
Fructose+mannose+glucose+Galactose+lactose	2.67 ± 0.71	2.25 ± 1.32	3.56

a Refers to the first binding step in Equation 1. Unit: number of modifications per αSN.

b Refers to the extra cooperativity of the second binding step in Equation 1.

c Refers to the second binding step in Equation 1. Errors estimated to around 50%. Unit: number of modifications per αSN.

In addition to the *level* of glycation, the *type* of glycation was also important for the degree of elongation inhibition. MGO and ribose modification both reduced the elongation rate to ∼10 to 20% of that of unmodified αSN, with MGO tending to slightly lower values in the minimum according to the fitted curve. In contrast, the less reactive glycation agents (fructose, mannose, glucose, galactose, and lactose) had much more limited effects on elongation rates. When the data for these five agents were combined into one set (which is reasonable given their generally similar behavior—Fig. 4*C*), it was possible to fit them to Equation 2 which reveals a reduction in elongation rates to ∼60% at the lowest rates. Clearly, these modifications “punched below their weight”, *i.e.*, they were not nearly as efficient as MGO and ribose in impeding αSN elongation per unit modification of αSN monomers (Fig. 4*D*). When combined with their much lower chemical reactivity toward αSN, this made them poor modulators of αSN elongation in particular and fibril formation in general.

### Glycation affects seeding in a cell model

**Figure 5:**
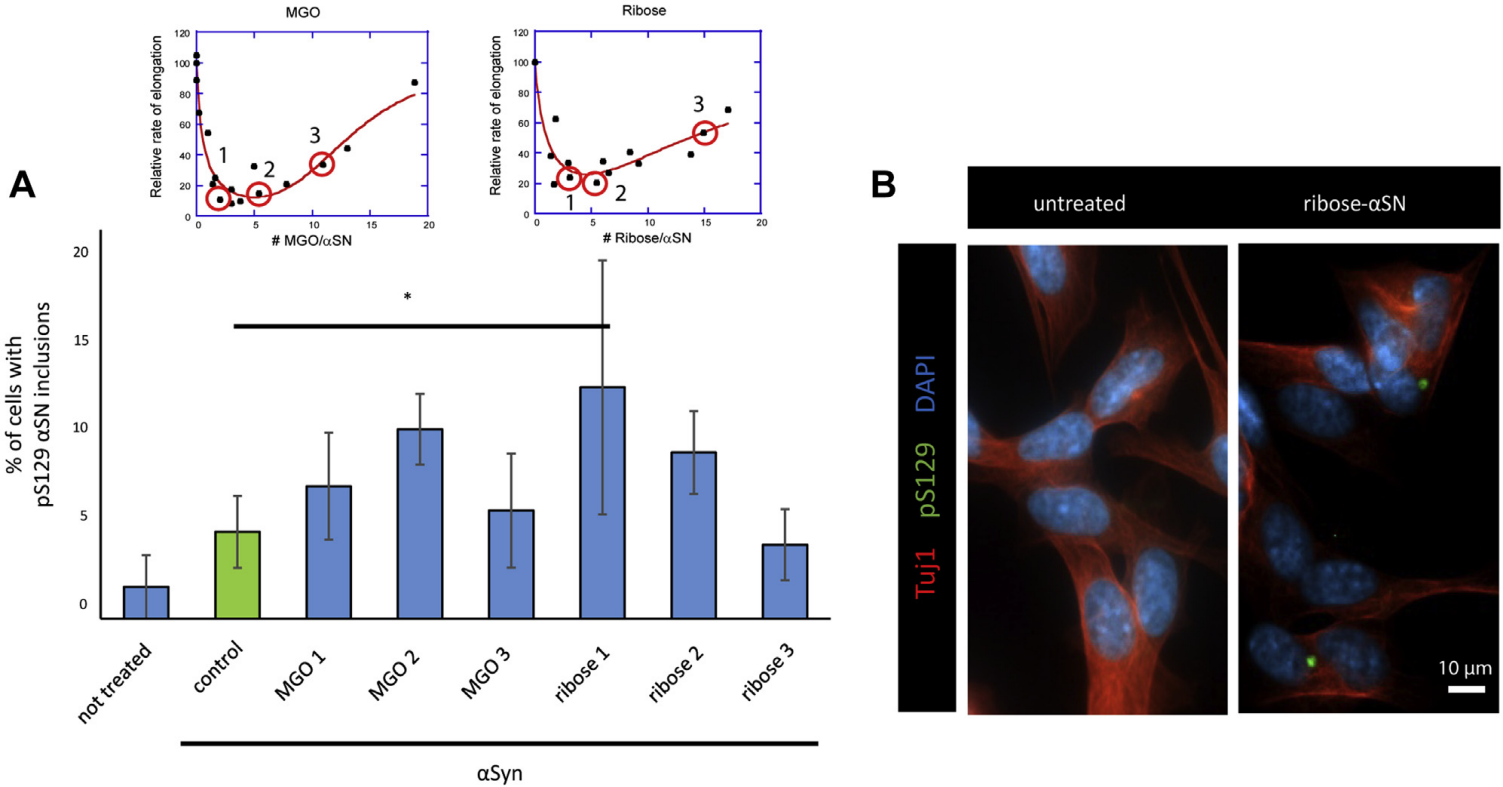
**Glycated αSN seeds aggregation in cells. SH-SY5Y cells with a Tet-Off cassette for regulating the inducible expression of WT αSN were differentiated for 4 days with retinoic acid.** Cells were then treated for another 4 days with 100 nM of the various αSN preparations and subjected to immunocytochemistry. The fraction of cells harboring pS129 positive inclusions (*B*) was quantified (*A*). *Red circles* in the inset specified with a number show the samples tested in the experiment. Insets in (*A*) are identical to Figure 4, *A* and *B* and were placed here for a better clarity. Scale bar: 10 μm. Statistical significance was assessed *via* ANOVA with a post hoc Tukey. ∗*p* < 0.05. αSN, α-synuclein.

We then tested the effect of MGO- or ribose-treated αSN on αSN seeding in SH-SY5Y cells inducibly expressing WT αSN. This cell model is widely used in the PD field (59, 60). αSN was modified with MGO or ribose to different extents (quantified by MS) and then added to cells. We quantified the fraction of cells containing phosphorylated Ser129 (pS129) αSN, since these inclusions are used as hallmark of PD-associated αSN pathology (61). No cytotoxicity was observed with the concentrations used (Fig. S7). Strikingly, the ribose-treated αSN that was shown to harbor less than five glycation residues per molecule (which have the greatest inhibitory impact on αSN fibril formation) induced significantly increased formation of pS129 αSN inclusions (Fig. 5, *A* and *B*).

## Discussion

### αSN is differently glycated by distinct glycating agents

MGO, a by-product of glycolysis, is highly reactive and is considered the most relevant glycating agent present in the cell (11). Therefore, it is often used as a model glycating agent. Other naturally occurring metabolites in living cells, including glucose, ribose, mannose, and fructose, have been far less studied in the context of αSN glycation. In this study, we set out to systematically compare the effects and mode of action of different sugars and potential glycating agents on αSN. We found that glycation rates for MGO and ribose resemble Michaelis–Menten kinetics that reflects the concentration dependence of the reaction which reaches saturation at high concentrations of glycating agent. Although the other glycating agents do not show evidence of saturation, this reflects their lower reactivity and suggests they are likely to saturate at higher (but physiologically unrealistic) concentrations. Based on initial rate measurements, we can classify the glycating agents into three groups with high (MGO), moderate (ribose), and low (mannose, glucose, galactose, lactose, and fructose) reactivity. Sucrose showed no detectable reactivity. Several studies have investigated the reactivity of glycation agents comparatively, but not quantitatively, and mostly using the compact globular protein albumin. These studies concluded, like we did, that dicarbonyl agents like MGO are the most reactive, followed by ribose and glucose (49, 62, 63). By combining fluorescence with MS, we were able to measure the kinetics of the reaction for each agent independently and, thereby, provide absolute rather than just relative values. Unsurprisingly, the dicarbonyl reagent MGO has the highest reactivity, ca. 30 times higher than the aldopentose ribose (which has one reactive aldehyde group). In turn, ribose is at least 25 times more reactive than the last group containing aldo-hexoses and keto-hexoses and the disaccharide lactose. In general, the reactivity of pentoses is higher than hexoses due to the instability of the furanose ring in some pucker configurations (64). Given the very similar levels of reactivity within the low-reactivity group, it is not surprising that the target protein or study conditions may change the internal order of reactivity within the group (62, 65).

Importantly, since glycation reactions are not specific for αSN, we expect our findings to be relevant for other proteins as well. On the other hand, given the amino acid composition of αSN, lacking arginine residues that are also a target for glycation, and with a high lysine content, likely leads to very particular glycation signatures.

### Glycation may affect αSN conformations through cross-links and electrostatics

Even though glycation changes the aggregation behavior of αSN, it does not affect the secondary structure of the monomers. In addition, a small amount of *paucimers* is formed by the most effective reacting agents (MGO and ribose). This might be explained by the ability of these agents to form crosslinks between two different amine groups. Formation of higher molecular weight species as a result of glycation cross-links has been reported for MGO and ribose (66, 67, 68, 69). Dimerization takes place in AGE products and some of the lysine-specific crosslinkers include crosslines, Fluorolink, glyoxal lysine dimers, methylglyoxal lysine dimers, and vesperlysines (Fig. S8). In addition, glycation of αSN may affect the transient interactions between αSN’s cationic N terminal and highly anionic C-terminal region by removing Lys’ positive charge. Thus, glycated αSN has a more negative zeta potential than unmodified αSN (32). Unsurprisingly, the removal of positive charges also profoundly reduces the ability of αSN to interact with anionic lipids. The faster mobility of MGO-modified αSN in native-PAGE may similarly reflect a decreased compactness of the protein because of changes in such transient intramolecular interactions.

### Glycated αSN suppresses elongation, probably by binding and blocking further incorporation

Our studies on the impact of glycated αSN on αSN fibril formation highlight the elongation step as the main target of glycated αSN. Elongation of a preexisting fiber requires monomers to first bind to the growing fibrils ends and then undergo a conformational change that allows it to incorporate into the fibril structure (70). Given the absence of glycated αSN in fibrils formed in their presence (even at high relative concentrations of glycated αSN), it seems clear that modified αSN is unable to incorporate stably into fibrils. Therefore, we propose that glycated αSN interferes with the elongation process by binding unproductively to the end of fibrils without fully integrating into the fibril structure or by binding in such a manner that its glycation groups block interactions with incoming monomers of αSN, thus preventing addition of new αSN monomers. This leads to an optimal level of glycation for fibril inhibition that balances affinity for the fibril ends with the inability to form a proper fibrillar structure.

A low number of modifications sufficed for optimal inhibition (4–5 per αSN for MGO and ribose and 2–3 for the hexoses and lactose). Although αSN is an intrinsically disordered protein without any persistent secondary structure, glycation is not evenly distributed among the different Lys groups. Human αSN expressed in yeast was mainly glycated in the N-terminal region (31); in contrast, an *in vitro* glycation study demonstrated a faster ribosylation in the NAC and C-terminal domains (residues 58, 60, 80, 96, 97, 102) rather than in the N terminus (67). Structural studies on αSN amyloid fibrils revealed several salt bridges involving K45, K58, and K80, glycation of which will obviously compromise fibril formation (71, 72). Modification of some specific Lys residues will block fibrillization to a greater extent than others, and the pattern of glycation may also play a role in how glycation affects fibrillization.

An important conclusion is also that not only the level of glycation but also the type of glycation is important for the efficiency of inhibition. Thus, while MGO reduces elongation by 90%, the corresponding numbers are ca. 70% for ribose and 40% for the remaining sugars. Counterintuitively, the smallest agent (MGO) has the largest effect, while the effect of the sugars remains difficult to rationalize by size or chemical structure. A glycosylation study on αSN with similar compounds (GlcNAc, GalNAc, Glc, and Man) showed that even minor chemical differences between these compounds lead to different levels of inhibition of fibril formation as well as formation of different fibrils (73). It should also be remembered that AGE products are tremendously diverse (comprising hundreds of different compounds, only a few of which are shown in Fig. S8) and produce different end products, even for similar agents like glucose and fructose (50, 74, 75, 76).

Using a cell-based model of synucleinopathy, we found that treatment with glycated αSN preparations lead to the formation of pS129-positive inclusions. However, treatment with MGO-αSN and some ribose-αSN preparations were not statistically different from unmodified αSN preparations. Studies showed that recombinant αSN is taken up by neuronal cells *via* different mechanisms including receptor-mediated endocytosis (77, 78). Despite some controversy regarding the mechanisms through which different αSN species are taken up and cleared in the cell, it seems that αSN monomers, oligomers, and fibrils follow different pathways. We showed in a previous study that MGO treatment of cells leads to impaired membrane binding and clearance of αSN (31). Additional studies will specifically focus on evaluating the effect of glycation on αSN uptake and clearance in neuronal cells.

### Implications for aggregation *in vivo*

What are the implications for our understanding of PTMs as a means of regulating or modifying αSN properties in the cell? Glycation distinguishes itself from other PTMs as a nonenzymatic and typically irreversible reaction. More carefully controlled PTMs such as ubiquitination, SUMOylation, and/or acetylation (all targeting Lys groups) also tend to decrease the fibrillization propensity of αSN (79, 80). While the importance of modification intensity has not been in focus in these studies, optimal levels of modifications are likely to operate in these cases as well, although these levels are likely to vary between glycation agents. Thus, ubiquitinylation or SUMOylation are likely to show much lower optimal levels of inhibition. In any case, these modifications occur more specifically and in a more well-regulated fashion and can occur at significantly lower levels, for example αSN is only acetylated at two out of 15 possible positions (81). Optimal levels of modifications are also likely to operate for other aggregation-prone proteins. While, to our knowledge, no studies addressed this, one study on tau protein showed that acetylation of tau by an acetyl transferase enzyme inhibits the fibrillation of (unmodified) tau to a greater extent than nonenzymatically acetylated tau does (82).

## Experimental procedures

### Materials

MGO, D-ribose, and D-fructose were obtained from Sigma; D-mannose, D-galactose, and D-sucrose from Duchefa Biochemie; D-glucose from Fisher Scientific; and D-lactose from Fluka Chemie. All reagents were analytical grade.

### Protein expression and purification

Human WT αSN was expressed in *E. coli* BL21 (DE3) cells using autoinduction media as described (83). αSN was purified on HiTrap Q HP anion exchange column in pH 7.4 after an acid treatment (pH 3.5) of crude protein. Purified protein was dialyzed in MQ water, lyophilized, and kept at −80 °C until further use.

### Glycation of αSN

Glycating agents and lyophilized αSN were dissolved in PBS (13 mM phosphate, 137 mM NaCl, 3 mM KCl, pH 7.4) and filtered with a 0.2 μM nitrocellulose membrane syringe filter. αSN concentrations were measured by Nanodrop 1000 spectrophotometer (Thermo Scientific) using a theoretical extinction coefficient at 280 nm of 5960 M^−1^ cm^−1^. Final concentrations of 100 μM αSN, 2 mM EDTA, 0.05% NaN_3_, and 0.5 to 1500 mM of glycating agents (see Results for details) were prepared in 1.5 ml microtubes and incubated at 37 °C. For kinetic studies of glycation, the prepared samples were transferred to a black polystyrene 96-well plate, and the fluorescence of AGE products were monitored by a plate reader (Thermo Scientific Varioskan Flash). AGE products were excited at 335 nm, and the emission was measured at 460 nm, with 40 μs integration time, every 30 min, without agitation. After incubation, excess glycating agents were removed using a PD 10 desalting column (Sigma Aldrich).

In glycation experiments performed with constant shaking, αSN and glycating agents (see Results for details) in PBS with 3.7 mM EDTA were filtered with a 0.2 μM nitrocellulose membrane syringe filter and incubated for 5 days at 37 °C under constant agitation (300 rpm) in low binding tubes (Corning Incorporated).

### CD spectroscopy

Far-UV CD spectra were recorded from 250 to 195 nm on a J-810 spectrophotometer (Jasco Spectroscopic Co Ltd) using 1 mg/ml αSN monomer in a 0.1 mm cuvette, 1 nm bandwidth, and steps of 1 nm per 0.5 s. Data are averages of two scans.

### MALDI-TOF MS

Desalted αSN from glycation experiments was diluted in 0.1% TFA and mixed 1:1 (vol:vol) with 2,5-dihydroxyacetophenone (0.1 M in 20 mM ammonium dihydrogen citrate and 75% (v/v) EtOH) (84). The material was spotted onto a stainless-steel target and allowed to dry. The spectra were recorded in positive and linear mode using an AutoFlex Smartbeam III instrument (Bruker) calibrated by external calibration (peptide calibration standard I, Bruker Daltronics). The centroid masses determined were evaluated using the GPMAW software (www.gpmaw.com).

### ThT fibril formation assays

The kinetics of fibril formation were measured by monitoring ThT fluorescence emission on a TECAN Infinite M200 plate reader at 37 °C using excitation at 448 nm and emission at 485 nm. Each sample contained a fixed amount of unmodified αSN and various amount of desalted glycated αSN as described in Results. Nonseeded experiments were performed with 10 min of shaking (300 rpm) in each 12 min measurement interval with 3-mm glass beads. Seeded experiments without shaking were performed in the presence of 5% of unmodified freshly sonicated seeds (probe sonicated for 15 s). All ThT experiments were performed with 1 mg/ml unmodified αSN unless otherwise stated. For seeded experiments with shaking, 100 μl of reaction mixtures were pipetted in black 96-well plates in triplicate (Corning Incorporated). The composition of the reaction mixtures was 150 mM NaCl, 1 mM EDTA, 10 μM ThioT, 70 μM SDS, 10 μg/ml of monomeric αSN, and 1 μg/ml glycated αSN preparations. Plates were subjected to 250 cycles of 1 min shaking at 432 rpm, followed by 2 min incubation.

### Cell cultures

Human neuroblastoma (SH-SY5Y) cells conditionally expressing human WT αSN with a Tet-Off cassette (59) were grown in RPMI supplemented with 1 μM doxycycline (to suppress αSN expression), 10% FBS, and 1% penicillin/streptomycin at 37 °C and 5% CO_2_. The cells were seeded in 6-well plates and differentiated for 1 day after plating using 10 μM retinoic acid in RPMI supplemented with 1% FBS and 1% penicillin/streptomycin, at 37 °C and 5% CO_2_ for 4 days. Media was changed every second day. Following 4 days of differentiation, cells were treated with 100 nM of the respective glycated αSN species for 4 days. The αSN species were prepared with constant shaking as described above. αSN treated the same way but without glycating agents was used as control. Cell viability was analyzed using a ToxiLight kit (Lonza) according to the manufacturer’s instructions.

### Immunoblotting

For native PAGE, 5 μg αSN was loaded onto a precast nondenaturing gel (Serva) and transferred to a nitrocellulose membrane. For SDS-PAGE, αSN was boiled in Laemmli buffer at 95 °C for 5 min, loaded to 12% Bis-Tris-polyacrylamide gels, transferred to nitrocellulose membranes, and processed with standard techniques. As primary antibody, we used anti-αSN1 (BD Biosciences).

### Immunofluorescence analyses

For immunofluorescence, cells were cultured on glass coverslips. Before fixation, cells were washed with PBS and incubated with trypsin for 5 min at 37 °C to remove residual protein species. 4% paraformaldehyde was used to fix the cells (20 min). Permeabilization was performed with 0.5% Triton X-100 at room temperature for 20 min. The cells were then blocked with 1.5% bovine serum albumin for 2 h. After blocking, cells were incubated overnight at 4 °C with the corresponding primary antibodies diluted in 1.5% BSA. The antibodies used were anti-Neuronal Class III Tubulin (Tuj1 – 1:3000, Covance, mouse host) and a pS129 (1:2000, Abcam51253, rabbit host). The cultures were washed three times with PBS and incubated for 2 h with secondary antibodies: Alexa Fluor 568-conjugated donkey anti mouse (1:2000, Life Technologies-Invitrogen) and Alexa Fluor 488-conjugated donkey anti rabbit (1:2000, Life Technologies-Invitrogen). Finally, cells were stained with DAPI for 5 min and cover slipped with Mowiol.

### Statistical analysis

All data were obtained from at least three independent experiments and are expressed as mean values ± standard deviation (SD). Two-group comparisons were performed with Student’s *t* test, and multiple-group comparisons were performed using ANOVA with post hoc Tukey. Differences were considered as statistically significant at *p* < 0.05. Statistical analyses were performed in Excel.

## Data availability

Data are contained within the article and its Supporting information.

## Supporting information

This article contains supporting information (85, 86).

## Conflict of interest

The authors declare that they have no conflicts of interest with the contents of this article.
