# Supplementary material for: Glycation modulates alpha-synuclein fibrillization kinetics: A sweet spot for inhibition
Source: J Biol Chem. 2022 Mar 18;298(5):101848. doi: 10.1016/j.jbc.2022.101848 (PMC9034100; doi:10.1016/j.jbc.2022.101848)
Supplement: Supplemental Figures S1–S8 [file mmc1.docx]

**Glycation modulates alpha-synuclein fibrillization kinetics: a sweet spot for inhibition**

Azad Farzadfard, Annekatrin König, Steen Vang Petersen, Janni Nielsen, Eftychia Vasili, Antonio Dominguez-Meijide, Alexander K. Buell, Tiago Fleming Outeiro and Daniel E. Otzen

**Supplementary information**


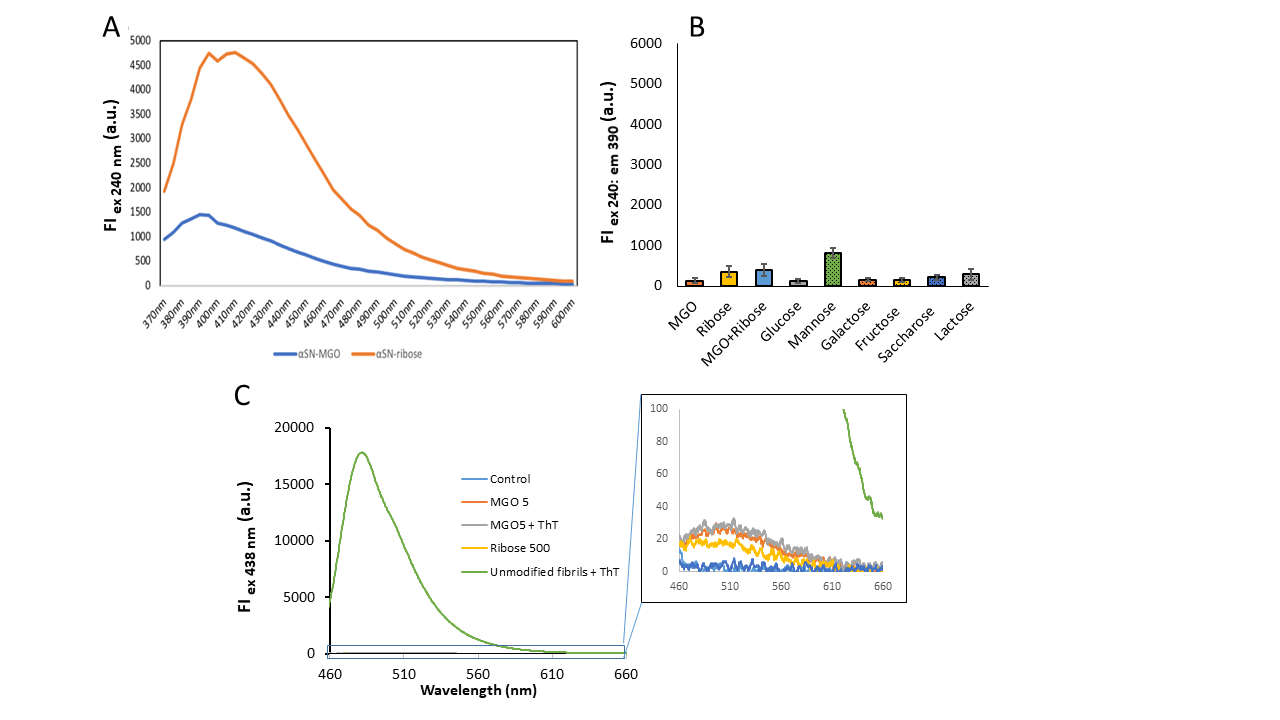


**Figure S1.** (A) Increase in fluorescence of αSN in the presence of glycating agents. 100mM αSN was incubated with 5mM MGO or 0.8M ribose for 5 days at 37°C under constant agitation (300rpm). Fluorescence was measured by excitation at 240nm. (B) Glycating agents do not produce fluorescence on their own. Glycating agents (5mM MGO, 0.8M ribose, 1M glucose, 1M mannose, 0.25M galactose, 2M fructose, 1.25 M saccharose, 0.5M lactose) were incubated for 5 days at 37°C in the absence of αSN under constant agitation (300rpm). Fluorescence was measured by excitation at 240nm and emission at 390nm. (C) Fluorescence spectra after excitation at 438 nm were measured to compare the emission intensity of different species used in ThT assay. ThT fluorescence intensity in the presence of the same amount of fibrils (40 µM) overwhelmingly eclipses fluorescence from glycated αSN.


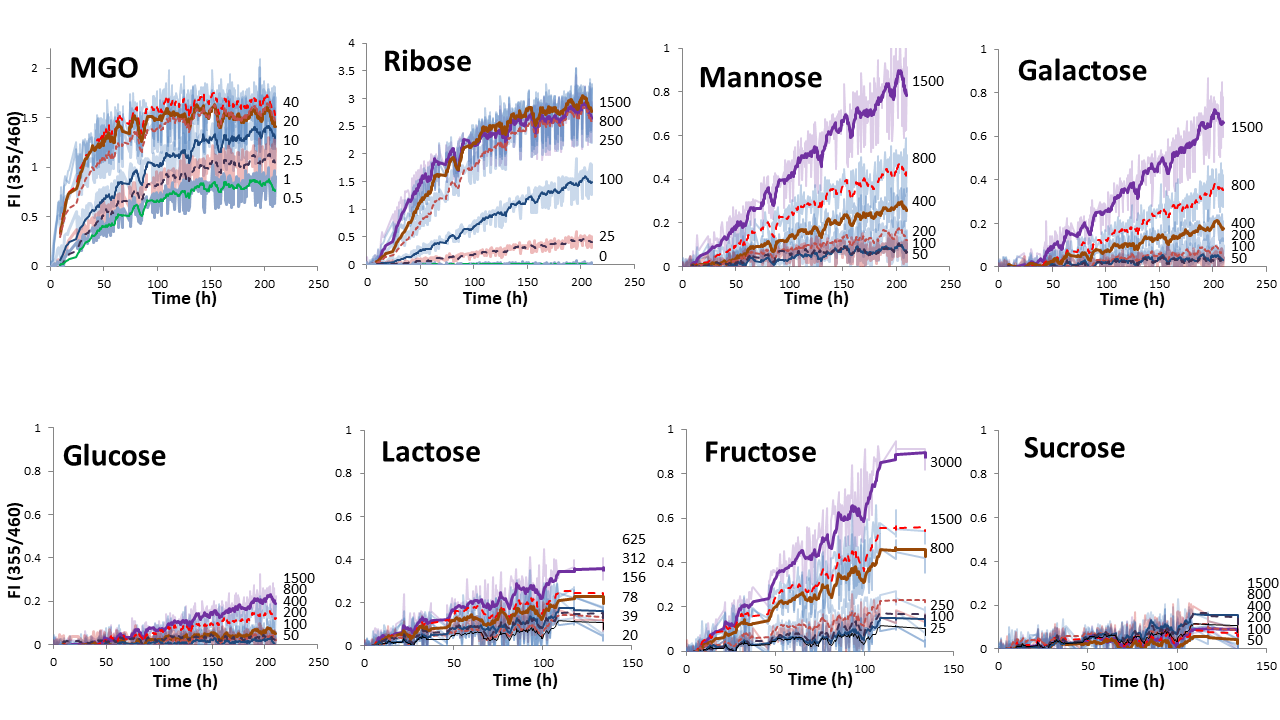


**Figure S2.** Kinetics of αSN glycation reaction measured by fluorescence (excitation/emission: 355/460 nm). Concentrations of each agent is provided in mM besides the corresponding curve. All data were collected under identical conditions in the same plate.


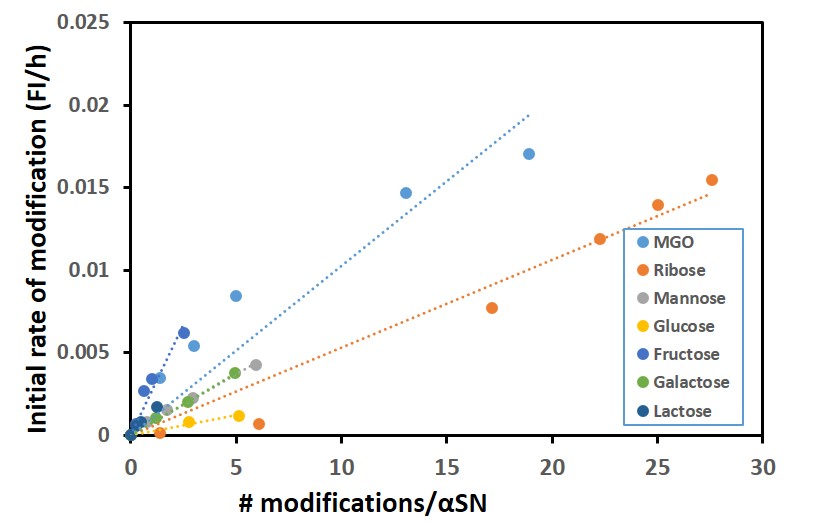


**Figure S3.** The initial rate of AGE fluorescence signal increase (based on the emission at 460 nm) correlates with the extent of modification of αSN *(*number of glycation groups attached to αSN measured by MALDI-TOF MS). However, different agents show different slopes, implying that modification by different agents leads to different levels of fluorescence.


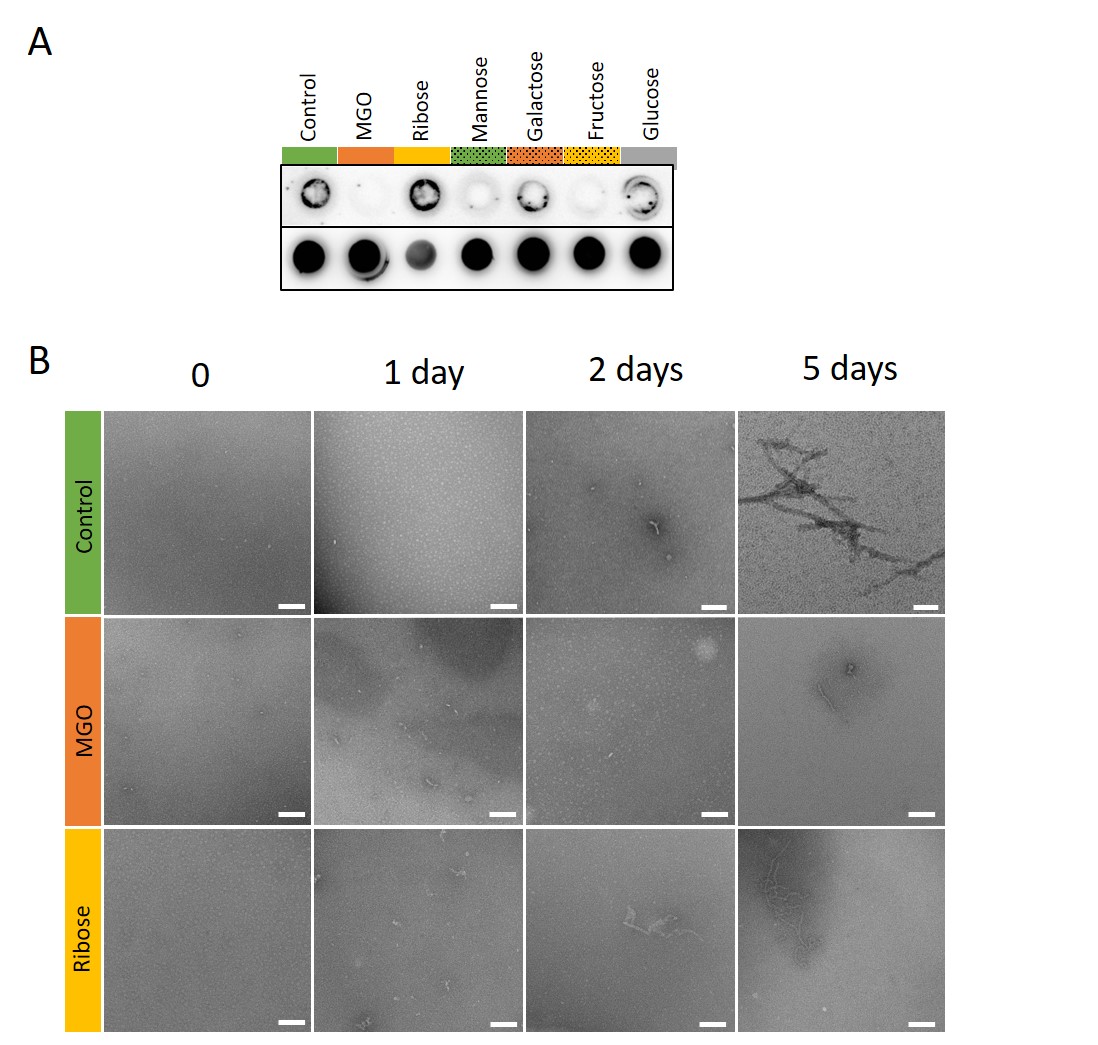


**Figure S4**. Solubility of glycated αSN. (A) 10µg of glycated αSN was spotted onto a 0.2um cellulose acetate membrane where only insoluble material was retained (upper panel). As control, a nitrocellulose membrane (where all material was retained) was used (lower panel). αSN specific antibodies (BD Transduction Laboratories) were used to detect αSN. Glycated αSN was prepared as in Fig. 1A, *i.e.* 100μM αSN was incubated alone or with different glycating agents (5mM MGO, 0.8M ribose, 1M glucose, 1M mannose, 0.25M galactose, 2M fructose, 1.25 saccharose, 0.5M lactose) for 5 days at 37°C under constant agitation (300rpm). αSN specific antibodies (BD Transduction Laboratories) were used to detect αSN. (B) Time series of TEM images of αSN species obtained in Fig. 3B. Scale bar: 200nm.


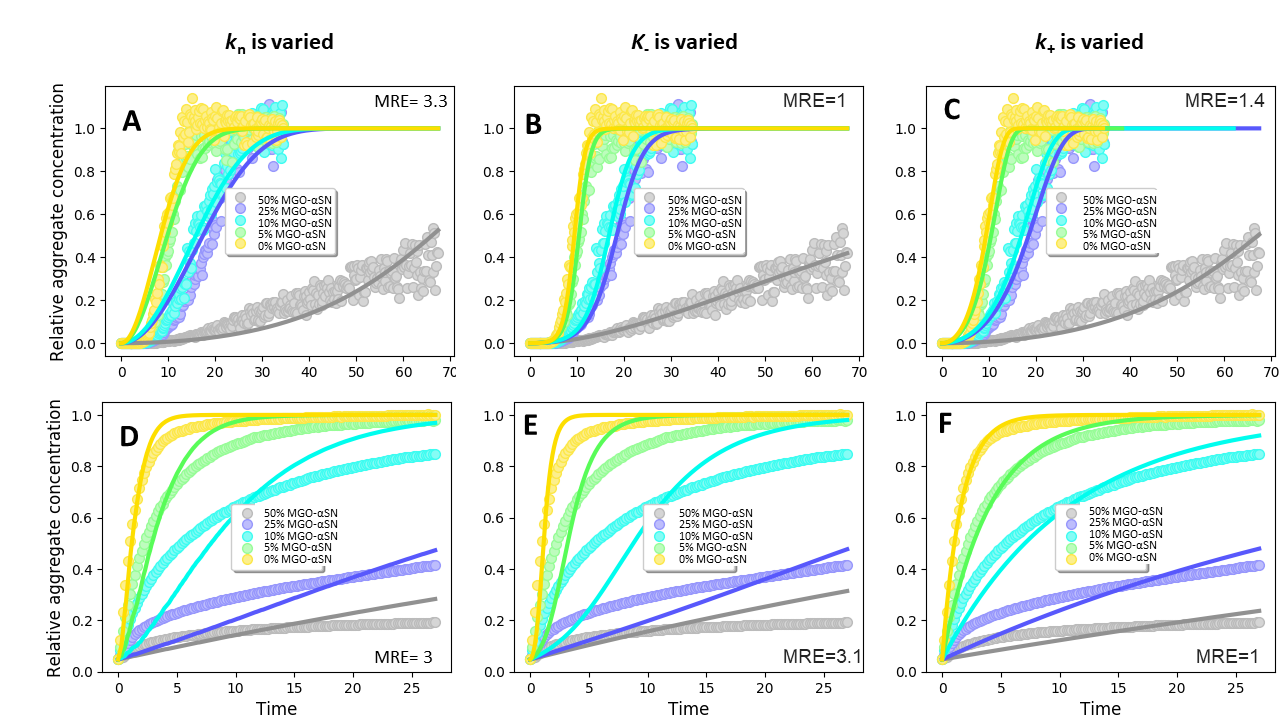


**Figure S5**. Amylofit analysis of the kinetic data in Fig. 3CD (fibril formation of 1 mg/ml unmodified αSN in the presence of 0-0.5 mg/ml (*i.e.* 0-50%) MGO-modified αSN, either in the absence (A-C) or presence (D-F) of 5% seeds). Data fitted to a model involving primary nucleation (*k_n_*), elongation (*k_+_*) and fragmentation (*k_-_*), in which only one parameter (indicated at the top of each column) is allowed to vary with the amount of added MGO-αSN; all other parameters are constrained to one global fit. Mean Squared Residual Error (MRE) normalized relative to the lowest MRE value for each of the two sets of measurements.


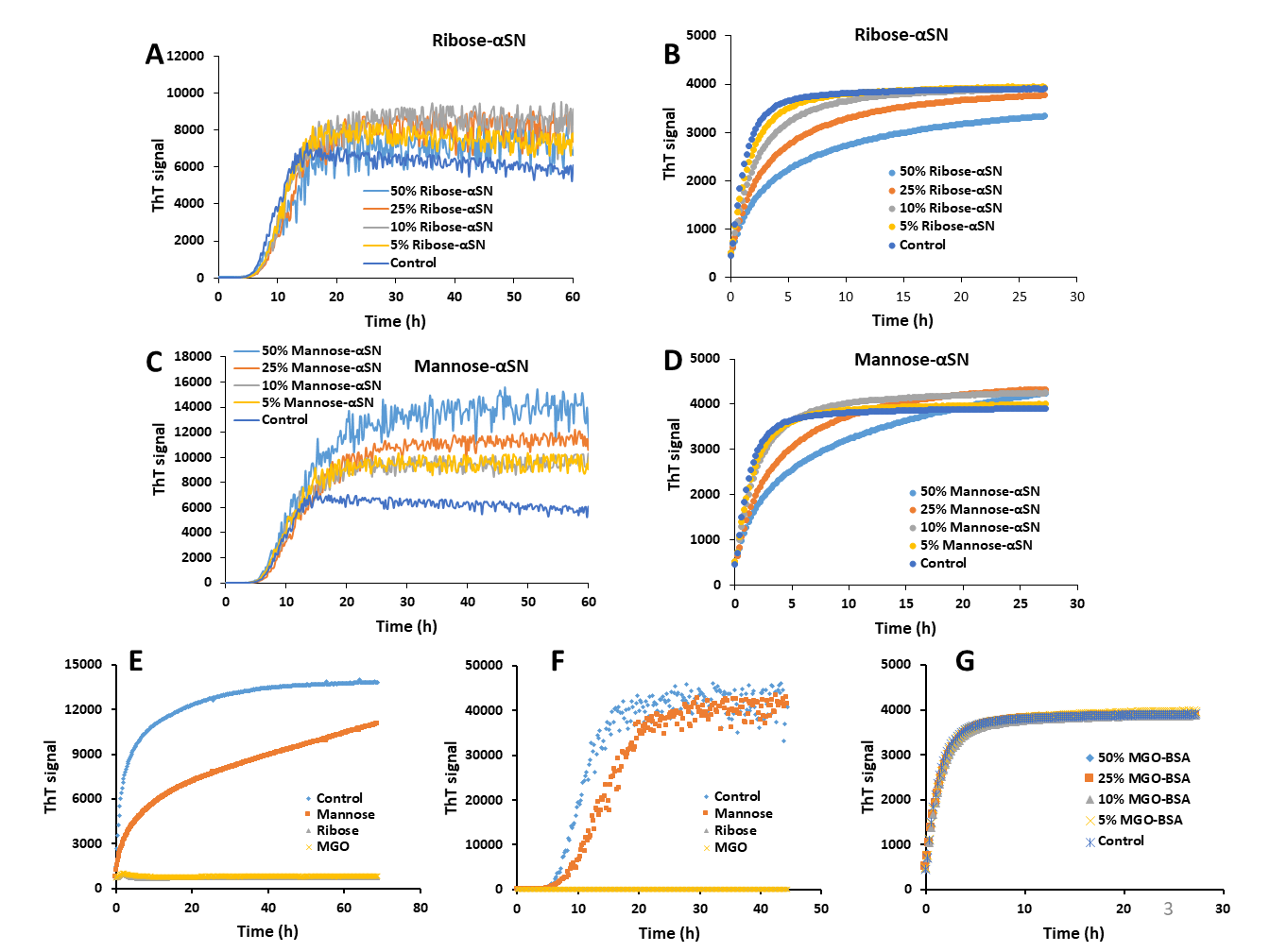


**Figure S6.** A-D) Fibril formation kinetics of 1 mg/ml unmodified αSN in the presence of 0-0.5 mg/ml (*i.e.* 0-50%) modified αSN without seed (A, C) and with 5% seed (B, D). E-F) Fibril formation kinetics of glycated αSN incubated with unmodified seeds (E) and without seeds (F) after 5 days incubation with a glycation agent (5 mM MGO, 800 mM ribose or 800 mM mannose). MGO-, ribose-, and mannose-modified αSN contain 7, 15, and 2 modifications according to MS. G) Glycated albumin does not influence the seeded fibril formation of unmodified αSN.

**Figure S7.** Viability of SH-SY5Y cells after treatment with 100nM of αSN preparations was analyzed using a Toxilight cytotoxicity assay. Viability was normalized to untreated control.

**
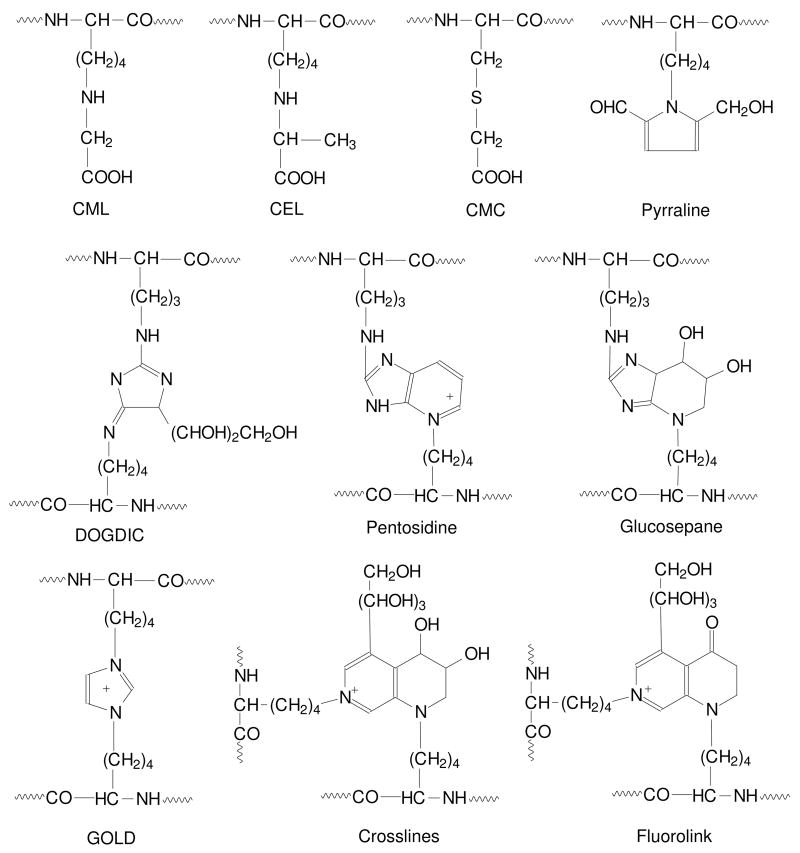
**

**Figure S8.** Chemical structure of lysine-mediated AGEs and crosslinks. CML (*N^ε^*-(carboxymethyl)lysine), CEL (*N^ε^*-(carboxylethyllysine)), GOLD (glyoxal lysine dimer), MOLD (methylglyoxal-lysine dimer), GLAP (glyceraldehyde-derived pyridinium compound)

Structures obtained from:

1. Nagai, R., Murray, D. B., Metz, T. O., and Baynes, J. W. (2012) Chelation: a fundamental mechanism of action of AGE inhibitors, AGE breakers, and other inhibitors of diabetes complications, *Diabetes* *61*, 549-559.
2. Grillo, M. A., and Colombatto, S. (2008) Advanced glycation end-products (AGEs): involvement in aging and in neurodegenerative diseases, *Amino Acids* *35*, 29-36.
